# Digital Control of Multistep Hydrothermal Synthesis by Using 3D Printed Reactionware for the Synthesis of Metal–Organic Frameworks

**DOI:** 10.1002/anie.201810095

**Published:** 2018-11-21

**Authors:** Chang‐Gen Lin, Wei Zhou, Xue‐Ting Xiong, Weimin Xuan, Philip J. Kitson, De‐Liang Long, Wei Chen, Yu‐Fei Song, Leroy Cronin

**Affiliations:** ^1^ Beijing Advanced Innovation Centre for Soft Matter Science and Engineering Beijing University of Chemical Technology Beijing 100029 P. R. China; ^2^ School of Chemistry The University of Glasgow Glasgow G12 8QQ UK; ^3^ State Key Laboratory of Chemical Resource Engineering Beijing University of Chemical Technology Beijing 100029 P. R. China

**Keywords:** 3D printing, hydrothermal synthesis, metal–organic frameworks, multi-step reactions, polyoxometalates

## Abstract

Hydrothermal‐synthesis‐based reactions are normally single step owing to the difficulty of manipulating reaction mixtures at high temperatures and pressures. Herein we demonstrate a simple, cheap, and modular approach to the design reactors consisting of partitioned chambers, to achieve multi‐step synthesis under hydrothermal conditions, in digitally defined reactionware produced by 3D printing. This approach increases the number of steps that can be performed sequentially and allows an increase in the options available for the control of hydrothermal reactions. The synthetic outcomes of the multi‐stage reactions can be explored by varying reaction compositions, number of reagents, reaction steps, and reaction times, and these can be tagged to the digital blueprint. To demonstrate the potential of this approach a series of polyoxometalate (POM)‐containing metal–organic frameworks (MOFs) unavailable by “one‐pot” methods were prepared as well as a set of new MOFs.

Hydrothermal or solvothermal reactions are normally performed in a closed system at temperatures above the boiling point of the solvent at standard pressure.^1^, ^2^ Hydrothermal conditions have been widely used to access many classes of materials including the synthesis of metal–organic frameworks (MOFs),^3^ covalent organic frameworks (COFs),^4^ extended metal oxides^5^ and polyoxometalates (POMs).^6^ Also, to help speed up the discovery and optimization of new materials, high‐throughput hydrothermal methods which allow the systematic investigation of the reaction parameters have been developed.^7^, ^8^ In addition, continuous flow hydrothermal techniques have been introduced for scalable synthesis.^9^ Despite these advances, conventional hydrothermal syntheses are still usually carried out under one‐pot, single step conditions, which precludes the exploration of multi‐step reactions or more complex reaction conditions. This limits the ability to introduce synthetic complexity, whereby the composition of the reaction mixture can be changed under the reaction conditions without interruption. Some approaches to multi‐stage hydrothermal reactions do exist, however these are limited to bespoke designed flow systems which are beyond the capabilities and means of many researchers.^10^

We hypothesized that sequential hydrothermal syntheses could be achieved by creating reactors with internal geometries that allow the compartmentalization of different reaction mixtures, preventing their mixing until defined points in the synthesis. This approach leads to the possibility of “trapping” otherwise inaccessible reaction intermediates or unstable building blocks. With this in mind, we opted to build upon our recent work that used 3D printing to give architectural control of the reactor with the design and fabrication of reactionware.^11^, ^12^, ^13^, ^14^, ^15^ This is because we wanted to see if the production of bespoke hydrothermal reactors, with partitioned chambers, was possible. Traditional “one‐pot” hydrothermal techniques are limited to using a single reaction composition (*c*) which encompasses the sum of starting materials introduced at the start of the experiment, and this evolves through the course of the reaction. By introducing separate compartments to the reactor we introduce a simple way of separating the starting materials into two compositions (*c*_1_ and *c*_2_) which can evolve separately, in parallel until mixed at new time, *t_n_*. As such the mixture can produce a new composition (c*), unavailable to traditional approaches, and these new mixtures can then continue reacting until the end of the experiment (Figure 1 A).

**Figure 1:**
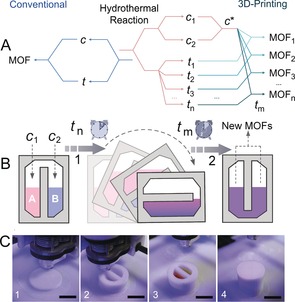
A) One step versus multistep hydrothermal synthesis in reactionware: *c* and *t* stand for composition and time parameters, respectively. B) Schematic illustration of the sequential multi‐stage hydrothermal synthesis in a 3D‐printed compartmentalized reactor. C) Photographs of the fabrication process of 3D printed reactor showing the sealing in of reagents (photos 3→4), scale bar 2 cm.

This architectural approach allows us to exert control over c* by varying *t_n_* to explore the time‐dependent kinetic studies on the “trapped” reaction intermediates.

In the first instance we applied this method for sequential hydrothermal synthesis, by geometrical control of the 3D printed reactor, to increase the synthetic parameter space and discover new materials unavailable by traditional techniques. To start with, we explored 8 new composition parameters of a set of possible MOFs from a network of 144 (3×6×8) possible reaction combinations using a 3D printed monolithic reactor (Figure 2). Then as an initial proof of concept, one of the eight new composition parameters was used to investigate the development of multi‐stage hydrothermal reactions with different POM clusters in a 3D printed compartmentalized reactor (Figure 1 B), leading to the formation of polyoxometalate–MOFs (POMOFs) **1** to **3** (Figure 3) which were unable to be produced by conventional “one‐pot” apparatus. To expand the scope of this reaction design and synthesis method using geometrical control further, time‐dependent studies of MOFs *c*_1_ and *c*_2_ on c* were explored and these gave rise to the discovery of the new MOF structures **9** to **11** (Figure 4).

**Figure 2:**
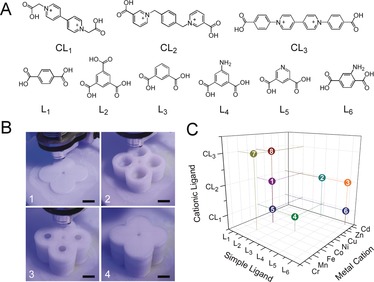
The screening of MOF structures used for multi‐stage reactions. A) The chemical structures of cationic ligands (CLs) and other organic ligands. B) Photographs of the fabrication process of 2×2 monolithic reactor. Scale bar: 1 cm. C) 8 new MOFs screened out from a network of 144 (3×6×8) possible combinations.

**Figure 3:**
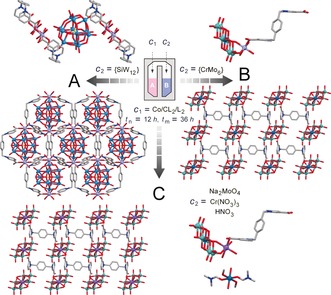
A) Top: the asymmetric unit of POMOF **1**; bottom: the crystal packing pattern of POMOF **1**. B) Top: the asymmetric unit of POMOF **2**; bottom: the crystal packing pattern of POMOF **2**. C) Right: the asymmetric unit of POMOF **3**; left: the crystal packing pattern of POMOF **3**. {SiW_12_}=K_4_[α‐SiW_12_O_40_]⋅17 H_2_O, {CrMo_6_}=Na_3_[CrMo_6_O_24_H_6_]⋅8 H_2_O. Co light blue, W turquoise, Mo aqua, Cr teal, Si yellow, Na purple, C grey, O red, N blue. H has been omitted for clarity.

**Figure 4:**
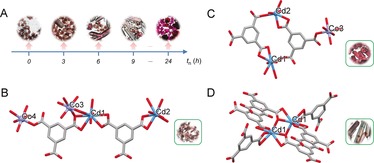
A) Photographs of the crystals formed by varying mixing time point *t_n_*. B) The asymmetric unit of MOF **9**. C) The asymmetric unit of MOF **10**. D) Dinuclear SBU of MOF **11**. Insets are photos of the crystals of MOFs **9** to **11**. Co light blue; Cd cyan; C grey; O red. H atoms, H_2_O and MV molecules have been omitted for clarity.

Prior to commencing the exploration of multi‐step hydrothermal reaction in the 3D‐printed reactors, the MOF components chosen for the multi‐step synthesis were screened in 2×2 array of approximately 3 mL capacity reaction chambers via traditional one‐pot reactions. Following our previous work,^13^ the reactors were constructed in a similar way by digital design in CAD software, followed by fabrication using the FDM deposition of polypropylene using a 3D printer. These new MOF candidates incorporate flexible cationic ligands (CLs) together with ditopic or tritopic ligands (L_1_–L_6_) into the frameworks (Figure 2 A). The introduction of CLs not only brings in flexibility because of their inherent diversity of conformations, but also allows the encapsulation of functional anionic clusters, such as POMs^16^, ^17^, ^18^, ^19^ within the frameworks of MOFs, which will be discussed further below, in relation to the multi‐step synthesis.

MOF structures **1** to **8** were prepared by optimizing the synthetic parameters using the 3D printed array reactors (Figure 2 B and Supporting Information Figure S2). All these compounds are new and feature open frameworks that are constructed from ligands L_1_–L_6_ and/or CLs with transition metal nodes, and the void is occupied by guest solvents and/or decomposed cations from CLs (Figure S4–S11). The formulation of MOFs **1** to **8** was determined on the basis of elemental analysis, IR spectroscopy, and thermogravimetric analysis (TGA). The phase purity of the bulk sample was established by comparison of its observed and simulated powder X‐ray diffraction (PXRD) patterns. The synthetic procedure, crystal data and crystal structures, and general characterization data of MOFs **1** to **8** can be found in Supporting Information including CCDC deposition numbers for the structural database.

Once a range of new MOFs compounds had been discovered in our 3D‐printed array of single chamber reactors, proof of concept experiments were carried out to explore multi‐step hydrothermal reactions. To perform the sequential hydrothermal synthesis, the reactor was designed with two partially connected chambers that would allow the compartmentalization of reaction components at the first stage, and the mixing of the different reaction intermediates at various points during hydrothermal process (Figure 1 B and Figure S1). The fabrication of the bespoke reactor was conducted on an Airwolf HD2x platform using polypropylene (PP). The starting reagents and solvents were manually added during a pre‐programmed pause when the printing was 80 % complete. The fabrication process was resumed and completed to give a hermetically sealed reactor, which was subsequently removed from the platform and transferred into an oven for multi‐stage hydrothermal reaction (Figure 1 C).

In the initial trial experiments, the ingredients of MOF **1** (Co/CL_2_/L_2_) were selected as compositional control parameters in *c*_1_ to explore multi‐step reactions with the Keggin‐type POM {SiW_12_} as the reagent in *c*_2_. The solutions of *c*_1_ and *c*_2_ remained in their respective chambers under hydrothermal conditions for 12 hours (*t*_n_), and then was mixed after turning the reactor upside down to produce a new composition c*, which was left undisturbed to continue reacting for another 36 hours (*t*_m_) under the same conditions. After cooling to room temperature, the reactor was opened using a drill and the reaction mixture was carefully extracted using a pipette. Single‐crystals of POMOF **1** were then obtained and analysed by X‐ray diffraction, infrared spectroscopy, thermogravimetric analysis and elemental analysis (see Supplementary Information). In the crystal structure of POMOF **1**, two cationic ligands CL_2_ coordinate to two Co ions to generate a cationic metallocycle (Figure 3 A). One {SiW_12_} cluster is sandwiched by two metallocycles via electrostatic interactions and multiple hydrogen bonds formed between both the terminal and bridging O atoms on {SiW_12_} and H atoms on the benzene ring of CL_2_. In addition, strong anion‐π interactions are also seen between the terminal O atoms of {SiW_12_} and aromatic ring of the linker with the anion‐to‐ring centroid contact of 2.943 and 3.006 Å, respectively (Figure S12). Varying the composition parameter *c*_2_ from Keggin anion to Anderson‐type {CrMo_6_} gave rise to POMOF **2**, featuring a 2D grid‐like framework built from the connection of central Co^2+^ ions with terminal O atoms of {CrMo_6_} and CL_2_ ligands (Figure 3 B). In contrast, owing to the strong electrostatic interactions between cationic ligands and POMs, control experiments showed that amorphous precipitates formed immediately once all the starting materials of *c*_1_ and *c*_2_ were added into a traditional “one‐pot” reactor. Such precipitates remained unchanged even after hydrothermal reactions (Figure S3). Therefore, these results unambiguously demonstrate the applicability of the two‐chamber rector for multi‐stage hydrothermal reaction and the advantage of this design towards preparation of new MOF structures that are inaccessible by conventional “one‐pot” approach.

Instead of using preformed POM clusters as a composition parameter *c*_2_, the compartmentalized reactor also allows the variation of *c*_2_ by in situ synthesis of POM clusters during the multi‐stage hydrothermal process. This, in principle, will provide more potential inputs that could further extend the synthetic parameter space. To do this, Na_2_MoO_4_ and Cr(NO_3_)_3_ were placed in one chamber to prepare {CrMo_6_} in situ before the turning point *t*_n_, and the resulting sequential synthesis led to a new compound POMOF **3**; this compound has similar 2D grid framework to POMOF **2**, and it is constructed by connection of Na^+^ ions with {CrMo_6_} and CL_2_ ligands, while free Cr^3+^ ions are located within the 2D open channels and exhibited octahedral geometries fulfilled by six O atoms from two DMF, two water, and two methanol molecules (Figure 3 C).

To further demonstrate the potential of the geometrical approach for discovery of new materials via the introduction of more synthetic parameters, time‐dependent and kinetically controlled hydrothermal reactions were performed by mixing the two compositions (*c*_1_ and *c*_2_) at different time points *t_n_* to produce a series of new compositions (c*), which can potentially result in different products and thus new materials. To this end, the reagent for MOFs **5** (Co/CL_1_/L_2_) and **6** (Cd/CL_1_/L_6_) were added into the two compartmentalized chambers to conduct multi‐stage hydrothermal reactions. As shown in Figure 4 A, various crystal combinations could be obtained by varying the mixing time points *t_n_*. When the two chambers were mixed at *t_n_*=0, only one type of crystals (MOF **9**) formed. Single‐crystal X‐ray analysis revealed that **9** is composed of a 3D network constructed by connection of Cd and Co centres with L_2_ ligands (Figure 4 B). Bridged by L_2_, a trinuclear Cd1‐Co3‐Cd1 secondary building unit (SBU) is generated and further linked with adjacent trinuclear SBU and Cd2 nodes to afford a highly‐connected 3D framework. The cationic ligands CL_1_ are thermally unstable and decompose into methyl viologen (MV) fragments,^20^ which serve as guest molecules to fill the cavities of MOF **9**. By carefully tuning the mixing point *t_n_* from 0 to 3 hours, a new type of compound with hexagonal crystals began to form in addition to MOF **9** and the yield gradually increased when *t_n_* was varied from 3 to 6 hours (Figure 4 A). Such crystals, denoted as **10**, were also found to possess a 3D network constructed by connection of Cd and Co centres with L_2_ ligands (Figure 4 C).

Two kinds of Cd centres and one type of Co centre are found in compound **10**. Cd1 and Cd2 are connected by **L_2_** to build a dinuclear SBU extending into a 2D framework along *a* axis, with the peripheral L_2_ further bound by Co3 to construct a 3D open framework. Similar to **9**, the open channel is filled by solvent and MV molecules. Further increasing *t_n_* to 9 hours afforded rod‐like crystals together with **9** and **10**. X‐ray analysis revealed that such rod crystals, named herein as MOF **11**, are only comprise eight‐coordinate Cd1 centres and L_2_. The basic SBU is based on a dinuclear motif built from two symmetry‐related Cd1 centres (Figure 4 D). Each SBU is surrounded by six **L_2_** and each **L_2_** connects with three SBUs, thus giving rise to a 3D open framework. A MV molecule is found to be located within the channels of compound **11**. Once the two compartmentalized reaction systems were mixed after 24 h of reaction, mixed crystals of **5** and **6** were obtained, indicating these MOF crystals have already formed after 24 h in separate chambers, and dominated as the stable species that cannot be accessible to either ligand or metal exchange.

These experiments clearly demonstrate that by mixing *c*_1_ and *c*_2_ at variable points *t_n_*, it is possible to exert control over the c* to create a whole new complex system that allows access to different reaction intermediates, leading to exploitation of new materials more easily and efficiently. It should be mentioned that MOFs **9** to **11** can be reproduced in large scale using traditional “one‐pot” method once the synthetic recipes are established by 3D‐printed multi‐stage reactions. However, to extend the time‐dependent synthetic strategy, similar procedures were also adopted for the discovery of new MOFs by replacing the initial parameters *c*_1_ and *c*_2_ with **4** (Co/CL_1_/L_4_) and **6** (Cd/CL_1_/L_6_), respectively. This gives different results compared to the approach using **5** and **6**, in which MOF **12** is obtained as the sole product irrespective of mixing points *t_n_*. MOF **12** features a 2D layer structure constructed from Cd^2+^ ions and L_4_, which is pillared by L_6_ to form box‐like 1D channel filled by MV molecules (Figure S13). A control experiment was done using “one‐pot” reactors to produce a mixture of MOF **12** and MOF **13**. X‐ray analysis showed that **13** is assembled from Cd ions and L_6_, and displays 1D zigzag chain structure. In contrast to **12**, the guest MV molecules are wrapped within the pitches between adjacent chains (Figure S14). These results indicate that the time‐dependent strategy has the potential to be used for manipulating the production distribution, and thus enable the selective formation of targeted product from a complex mixture.

In summary, we have demonstrated a new conceptual approach for the hydrothermal synthesis of compounds, controlled by geometrical design, and implemented using 3D printed reactionware for multi‐stage hydrothermal reactions. This 3D method enables us to divide the reaction control into more compositional parameters (*c*_1_, *c*_2_…*c_n_*), and also introduces a new time parameter *t_n_* for mixing *c*_1_ and *c*_2_ to produce c*. Thus, by increasing the number of parameters, we have shown it is possible to open up new areas of the synthetic space, which should allow researchers to search for new materials, impossible to make using conventional methods. Finally, the digital design of the reactors, coupled with tagging the synthetic meta‐data will lead to improved collaboration, and reproducibility of results. This will be made possible via development of a digital‐chemical code for hydrothermal synthesis we will develop in the future based upon the reactionware approach.^11^

## Conflict of interest

The authors declare no conflict of interest.

## Supporting information

As a service to our authors and readers, this journal provides supporting information supplied by the authors. Such materials are peer reviewed and may be re‐organized for online delivery, but are not copy‐edited or typeset. Technical support issues arising from supporting information (other than missing files) should be addressed to the authors.

Supplementary
