# Supplementary material for: Digital Control of Multistep Hydrothermal Synthesis by Using 3D Printed Reactionware for the Synthesis of Metal–Organic Frameworks
Source: Angew Chem Int Ed Engl. 2018 Nov 21;57(51):16716–20. doi: 10.1002/anie.201810095 (PMC6391986; doi:10.1002/anie.201810095)
Supplement: Supplementary file 1 — Supplementary [file ANIE-57-16716-s001.pdf]

Angewandte Chemie
Eine Zeitschrift der Gesellschaft Deutscher Chemiker

Supporting Information

Digital Control of Multistep Hydrothermal Synthesis by Using 3D Printed Reactionware for the Synthesis of Metal–Organic Frameworks

Chang-Gen Lin, Wei Zhou, Xue-Ting Xiong, Weimin Xuan, Philip J. Kitson, De-Liang Long, Wei Chen, Yu-Fei Song, and Leroy Cronin**

anie_201810095_sm_miscellaneous_information.pdf

General: All the chemicals were purchased from Alfa Aesar, Shanghai Energy Chemicals, or Beijing Chemical Works, and used as received. FT-IR spectra were recorded on a Bruker Vector 22 infrared spectrometer using KBr pellets. Thermogravimetric (TG) curves of all the MOFs were carried out on Mettler Toledo 1/110 SF under N_2 flow with a heating rate of 10 °C min^{-1}; while those of POMOFs were recorded on a TA Q500 V6.7 Build 203 instrument under N_2 flow with a heating rate of 10 °C min^{-1}. Elemental analyses were completed by using varioEL cube from Elementar Analysensysteme GmbH. Single-crystal X-ray diffraction data were collected at 180 K on a Rigaku SuperNova Atlas S2 diffractometer (λ ($Cu_{k\alpha}$) = 1.54178 Å) equipped with a graphite monochromator. Structure solution and refinement were carried out with SHELXS-2014[1] and SHELXL-2014[2]. CCDC 1861853-1861865 contain the supplementary crystallographic data for MOFs **1**-**13**, and CCDC 1861866-1861868 contain the data for **POMOFs 1**-**3**. These data can be obtained free of charge via www.ccdc.cam.ac.uk/data_request/cif. Powder X-ray diffraction data were recorded on a Rigaku XRD-6000 diffractometer under $Cu_{K\alpha}$ radiation (λ = 0.1542 nm) at a scanning rate of 2 ° min^{-1}. The cationic ligands 1,1'-biscarboxymethyl- 4,4'-bipyridium (**CL$_1$**),[3] 1,4-bis(pyridinil-3-carboxylato)-1,4-dimethyl- benzene (**CL$_2$**),[4] and 1,1'-bis(4-carboxyphenyl)-4,4'-bipyridium (**CL$_3$**)[5] were prepared according to published methods and fully characterized.

Digital Design of reactionware: The reactionware was designed in AutoCAD 2016 software (Autodesk Inc.), and the files were translated into 3D stl printer instruction files using open source Cura15 software (Ultimaker). The CAD design of the reactors is shown in Fig. S1 and S2. The CAD files were designed with a simple workflow in mind, that is the two-step synthesis in two separate reaction volumes that share a common head space. The concentrations of the reagents and the stoichiometry can be controlled by the digitally defined reactor volumes, and the number of steps can be controlled by the number of reactors interlinked into the device. For this work, everything was done manually but according to the precise methods outlined here, and hence could be made entirely digital to increase reproducibility.

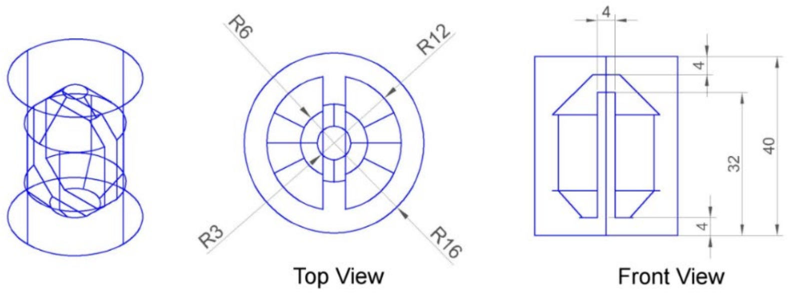

Figure S1. The CAD model of the compartmentalised reactor. Unit: mm.

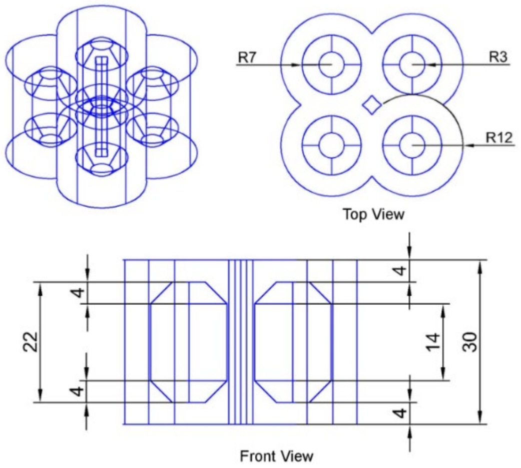

Figure S2. The CAD model of the 2 × 2 monolithic reactor. Unit: mm

3D printing of reactionware: 3D printing was achieved on Airwolf HD2x 3D printer using polypropylene (PP) supplied by Barnes Plastic Welding Equipment Ltd, Blackburn, UK. To prevent corners from lifting due to warping, PP was printed on a 5 mm thick PP sheet.

Syntheses: MOFs **1**-**8** were screened out and the synthetic parameters were optimized by using monolithic 2 × 2 array reactor. Here only the optimal synthetic conditions for large scale preparation in traditional apparatus are described.

1: $[Co_2(CL_2)_{0.5}(L_2)(OH)(H_2O)_2]\cdot 3.25H_2O$

$Co(NO_3)_2\cdot 6H_2O$ (58.2 mg, 0.2 mmol), CL_2 (105 mg, 0.3 mmol) and 1,3,5-benzene-tricarboxylic acid (21 mg, 0.1 mmol) were stirred in the H_2O (2 mL), DMF (4 mL), and methanol (2 mL). The mixture was heated to 85 ^oC for 48 h and then left to cool down to room temperature. Purple crystals were collected by filtration. FT-IR (KBr, cm^{-1}): ν = 3852 (s), 3744 (m), 3672 (s), 3648 (s), 3419 (w), 2360 (m), 1648 (w), 1559 (s), 1458 (s), 1385 (w), 1271 (s), 1129 (m), 994 (s), 945 (m), 900 (w), 812 (m), 756 (w), 619 (s), 533 (s), 477 (s). Elemental analysis for $C_{19}H_{22.5}Co_2NO_{14.25}$ (%) calcd: C 37.33, H 3.68, N 2.29; found: C 38.79, H 3.534, N 2.84.

2: $[Cd(CL_2)_{0.5}(L_4)(H_2O)]\cdot H_2O$

$Cd(NO_3)_2\cdot 4H_2O$ (30.8 mg, 0.1 mmol), CL_2 (42 mg, 0.12 mmol) and 5-aminoisophthalic acid (18.11 mg, 0.1 mmol) were stirred in H_2O (2.5 mL), DMF (5 mL), and ethanol (2 mL). The mixture was heated to 85 ^oC for 48 h and then left to cool down to room temperature. Yellow block crystals were collected by filtration. FT-IR (KBr, cm^{-1}): ν = 3852 (s), 3744 (m), 3672 (s), 3648 (s), 3419 (w), 2360 (m), 1648 (w), 1559 (s), 1458 (s), 1385 (w), 1271 (s), 1129 (m), 994 (s), 945 (m), 900 (w), 812 (m), 756 (w), 619 (s), 533 (s), 477 (s). Elemental analysis for $C_{18}H_{17}CdN_2O_8$ (%) calcd: C 43.05, H 3.39, N 5.58; found: C 45.62, H 2.753, N 5.99.

3: $[Cd(CL_2)_{0.5}(L_6)(H_2O)]$

$Cd(NO_3)_2\cdot 4H_2O$ (61.6 mg, 0.2 mmol), CL_2 (35 mg, 0.1 mmol) and 2-aminoterephthalic acid (18.11 mg, 0.1 mmol) were stirred in H_2O (4 mL), DMF (4 mL), and methanol(2.5 mL). The mixture was heated to 85 ^oC for 48 h and then left to cool down to room temperature. Yellow dendrite crystals were collected by filtration. FT-IR (KBr, cm^{-1}): ν = 3852 (s), 3744 (m), 3672 (s), 3648 (s), 3419 (w), 2360 (m), 1648 (w), 1559 (s), 1458 (s), 1385 (w), 1271 (s), 1129 (m), 994 (s), 945 (m), 900 (w), 812 (m), 756 (w), 619 (s), 533 (s), 477 (s). Elemental analysis for $C_{18}H_{15}CdN_2O_7$ (%) calcd: C 44.65, H 3.10, N 5.79; found: C 43.79, H 3.002, N 5.97.

4: $[MV]_{0.75}[Co_3H_{0.5}(L_4)_4(H_2O)_2]\cdot3H_2O$

$Co(NO_3)_2\cdot6H_2O$ (29.1 mg, 0.1 mmol), CL_1 (34.5 mg, 0.1 mmol), and 5-aminoisophthalic acid (18.1 mg, 0.1 mmol) were added to a mixed solvent of DMF (1.5 mL), methanol (3 mL), and H_2O (2 mL). The mixture was stirred 5 min until completely dissolved, then sealed in a 10 mL Teflon-lined steel reactor and kept at 85 °C for 72 h and then left to cool down to room temperature. Reddish brown crystals were collected. FT-IR (KBr, cm^{-1}): ν = 3744 (w), 3454 (m), 3358 (m), 3045 (m), 2971 (m), 2922 (m), 2359 (w), 1971(w), 1631 (m), 1577 (s), 1465 (m), 1375 (vs), 1104 (w), 1020 (w), 951 (w), 914 (w), 783 (m), 708 (m), 541 (w), 450 (w). Elemental analysis for $C_{41}H_{41}Co_3N_{5.5}O_{21}$ (%) calcd: C 43.79, H 3.65, N 6.85; found: C 45.41, H 3.083, N 7.33.

5: $[MV][Co_5(L_2)_3(HL_2)(OH)(H_2O)_6]\cdot7.5H_2O\cdot2DMF\cdot CH_3OH$

$Co(NO_3)_2\cdot6H_2O$ (43.65 mg, 0.15 mmol), CL_1 (51.75 mg, 0.15 mmol), and 1,3,5-benzenetricarboxylic acid (21 mg, 0.1 mmol) were added to a mixed solvent of DMF (2 mL), methanol (3 mL) and H_2O (1.5 mL). The mixture was stirred 5 min until completely dissolved, then sealed in a 10ml Teflon-lined steel reactor and kept at 85 °C for 72 h and then left to cool down to room temperature. Purple crystals were collected by filtration. FT-IR (KBr, cm^{-1}): ν = 3425 (m), 2924 (w), 1632 (s), 1563 (m), 1437 (s), 1370 (s), 1255 (m), 1186 (w), 1102 (w), 823 (w), 764 (w), 716 (m), 558 (w), 456 (w). Elemental analysis for $C_{55}H_{73}Co_5N_4O_{41.5}$ (%) calcd: C 37.63, H 3.99, N 3.19; found: C 39.42, H 4.217, N 3.38.

6: $[MV]_2[Cd_3(L_6)_{4.5}(H_2O)_2Cl]\cdot7.5H_2O$

$Cd(NO_3)_2\cdot4H_2O$ (54 mg, 0.175 mmol), CL_1 (34.5 mg, 0.1 mmol) and 2-aminoterephthalic acid (18.1 mg, 0.1 mmol) were added to a mixed solvent of DMF (2 mL), ethanol (1 mL), and H_2O (1 mL). The mixture was stirred 5 min until completely dissolved, then sealed in a 10 mL Teflon-lined steel reactor and kept at 85 °C for 72 h and then left to cool down to room temperature. Brown block crystals were collected. FT-IR (KBr, cm^{-1}): ν = 3433 (m), 3333 (m), 3113 (w), 3047 (w), 1630 (m), 1557 (vs), 1426 (s), 1373 (s), 1258 (m), 900 (w), 836 (m), 776 (m), 705 (w), 574 (w), 478 (w). Elemental analysis for $C_{60}H_{69.5}Cd_3N_{8.5}O_{27.5}Cl$ (%) calcd: C 41.80, H 4.03, N 6.91; found: C 42.09, H 3.973, N 6.87.

7: $[Co_3(CL_3)(L_2)_2(H_2O)_7]\cdot4H_2O$

$Co(NO_3)_2\cdot6H_2O$ (29.1 mg, 0.1 mmol), CL_3 (16 mg, 0.033 mmol), and 1,3,5-benzenetricarboxylic acid (7 mg, 0.033 mmol) were added to a mixed solvent of DMF (3 mL), methanol (1 mL), and H_2O (3 mL). The mixture was stirred 5 min until completely dissolved, then sealed in a 10 mL Teflon-lined steel reactor and kept at 80 °C for 48 h and then left to cool down to room temperature. Reddish brown crystals were collected by filtration. FT-IR (KBr, cm^{-1}): ν = 3743 (w), 3316 (m), 3116 (m), 2359 (w), 1612 (s), 1554 (s),1367 (s), 1106 (w), 1012 (w), 835 (w), 772 (m), 717 (m), 652 (m), 556 (w), 473 (w). Elemental analysis for $C_{42}H_{44}Co_3N_2O_{27}$ (%) calcd: C 42.51, H 3.71, N 2.36; found: C 43.09, H 3.613, N 2.51.

8: $[Mn_3(CL_3)_2(L_2)_2(H_2O)_5]\cdot9.5H_2O$

$MnCl_2\cdot4H_2O$ (19.8 mg, 0.1 mmol), CL_3 (16 mg, 0.033 mmol), and 1,3,5-benzenetricarboxylic acid (7 mg, 0.033 mmol) were added to a mixed solvent of DMF (3 mL), ethanol (1 mL), and H_2O (3 mL). The mixture was stirred 5 min until completely dissolved, then sealed in a 10 mL Teflon-lined steel reactor and kept at 80 °C for 48 h and then left to cool down to room temperature. Yellow crystals were collected by filtration. FT-IR (KBr, cm^{-1}): ν = 3270 (m), 3122 (m), 2281 (w), 1634 (m), 1607 (s), 1566 (s), 1488 (m), 1439 (m), 1380 (s), 1305 (w), 1227 (w), 1115 (w), 1009 (w), 878 (w), 837 (w), 780 (m), 696 (w), 623 (w), 535 (w), 468 (w). Elemental analysis for $C_{66}H_{67}Mn_3N_4O_{34.5}$ (%) calcd: C 48.50, H 4.10, N 3.43; found: C 49.65, H 3.961, N 3.61.

POMOF 1: $[Co_2(CL_2)_2(H_2O)_8(SiW_{12}O_{40})]\cdot 3DMF$

When the printing was paused at 80 % completion, 4 mL of the starting mixture of MOF **1** and 2 mL of $K_4[\alpha\text{-}SiW_{12}O_{40}]\cdot 17H_2O$ (334.0 mg, 0.1 mmol) in H_2O/DMF/methanol (v/v, 1/2/1) were added into the reaction chambers A and B, respectively. The printing was restarted and completed. The 3D printed reactor was transferred into an oven and heated at 85 °C for 12 h. Then the reaction systems were mixed by rotation and continued to react at the same condition for another 36 h. After cooling down to room temperature, block pink crystals were collected. FT-IR (KBr, cm^{-1}): ν = 3852 (s), 3744 (m), 3672 (s), 3648 (s), 3419 (w), 2360 (m), 1648 (w), 1559 (s), 1458 (s), 1385 (w), 1271 (s), 1129 (m), 994 (s), 945 (m), 900 (w), 812 (m), 756 (w), 619 (s), 533 (s), 477 (s). Elemental analysis for $C_{49}H_{69}Co_2SiW_{12}N_7O_{59}$ (%) calcd: C 14.77, H 1.74, N 2.41; found: C 14.95, H 1.73, N 2.53.

POMOF 2: $H[Co(CL_2)(H_6CrMo_6O_{24})]\cdot 4H_2O\cdot 2DMF$

When the printing was paused at 80 % completion, 4 mL of the starting mixture of MOF **1** and 2 mL of $Na_3[CrMo_6O_{24}H_6]\cdot 8H_2O$ (108.6 mg, 0.1 mmol) in H_2O were added into the reaction chamber A and B, respectively. The printing was restarted and completed. The 3D-printed reactor was transferred into oven and heated at 85 °C for 12 h. Then the reaction systems were mixed by rotation and continued to react at the same condition for 36 h. After cooling down to room temperature, block reddish-brown crystals were collected. FT-IR (KBr, cm^{-1}): ν = 3744 (s), 3418 (m), 3068 (s), 2362 (s), 1642 (w), 1606 (m), 1576 (m), 1495 (s), 1466 (m), 1386 (w), 1196 (m), 1133 (m), 1013 (m), 939 (m), 909 (m), 867 (m), 759 (m). Elemental analysis for $C_{26}H_{45}CoCrMo_6N_4O_{34}$ (%) calcd: C 19.16, H 2.47, N 3.44; found: C 19.36, H 2.669, N 3.13.

POMOF 3: $[Cr_{0.67}(H_2O)_2(CH_3OH)_2(DMF)_2][Na(CL_2)(H_6CrMo_6O_{24})]\cdot 4H_2O$

When the printing was paused at 80 % completion, 4 mL of the starting mixture of MOF **1** and 2 mL mixture of $Na_2MoO_4\cdot 2H_2O$ (511.2 mg, 2.10 mmol) and $Cr(NO_3)_3\cdot 9H_20$ (132.5 mg, 0.33 mmol) in H_2O (pH = 4.5) were added into the reaction chamber A and B, respectively. The printing was restarted and completed. The 3D-printed reactor was transferred into oven and heated at 85 °C for 12 h. Then the reaction systems were mixed by rotation and continued to react at the same condition for 36 h. After cooling down to room temperature, block red crystals were collected. FT-IR (KBr, cm^{-1}): ν = 3847 (s), 3272 (s), 3118 (s), 3052 (m), 2362 (s),1619 (m), 1563 (w), 1452 (m), 1426 (m), 1386 (w), 1302 (s), 1281 (s), 1214 (s), 1174 (m), 1137 (m), 1119 (s), 1047 (s), 933 (s), 911 (s), 892 (m), 792 (m), 766 (m), 651 (w), 583 (s), 415 (m). Elemental analysis for $C_{28}H_{56}Cr_{1.67}Mo_6N_4O_{38}Na$ (%) calcd: C 19.02, H 3.17, N 3.17; found: C 18.32, H 3.01, N 2.95.

Multi-stage hydrothermal reactions in 3D-printed compartmentalized reactors are as follows: 4 mL of the starting mixtures of two different MOFs was introduced into the reaction chambers A and B, respectively, when the printing was automatically paused at 80 % completion. The printing was resumed, and fabrication completed. The printed reactor was put into an oven and heated to hydrothermal conditions. Mixing the reaction systems was thereafter carried out at different points by turning the reactor upside down (*CAUTION*: Oven gloves are needed to prevent burns). Then the reactor was left under hydrothermal conditions until the reaction completed. The reaction mixture was carefully extracted by a pipette through the hole opened by a drill (*CAUTION*: Do operate in fume hood).

MOF 9: $[MV]_2[Cd_4Co_3(L_2)_6(H_2O)_{12}]\cdot 14H_2O$

FT-IR (KBr, cm^{-1}): ν = 3419 (m), 3053 (m), 1614 (s), 1561 (s), 1438 (s), 1374 (vs), 1222 (w), 1185 (w), 1106 (w), 937 (w), 826 (w), 768 (m), 726 (m), 534 (w), 452 (w), 407 (w). Elemental analysis for $C_{78}H_{98}Cd_4Co_3N_4O_{62}$ (%) calcd: C 34.54, H 3.62, N 2.07; found: C 35.32, H 3.673, N 2.16.

MOF 10: $[MV][Cd_4Co(L_2)_4(H_2O)_8]\cdot 6H_2O$

FT-IR (KBr, cm^{-1}): ν = 3442 (s), 2919 (m), 2850 (m), 2355 (w), 1614 (s), 1561 (s), 1437 (s) ,1375 (s), 1186 (w), 1108 (w), 943 (w), 830 (w), 771 (m), 725 (m), 523 (w), 464 (w), 416 (w). Elemental analysis for $C_{48}H_{54}Cd_4CoN_2O_{38}$ (%) calcd: C 34.78, H 3.28, N 2.70; found: C 34.02, H 3.431, N 2.05.

MOF 11: $[MV]_{0.5}[Cd(L_2)(H_2O)]\cdot 2.5H_2O$

FT-IR (KBr, cm^{-1}): ν = 3420 (s), 3060 (m), 2359 (w), 1642 (m), 1611 (s), 1562 (s), 1437 (s), 1374 (vs), 1191 (w), 1104 (w), 933 (w), 828 (w), 768 (m), 728 (m), 519 (w), 418 (w). Elemental analysis for $C_{15}H_{17}CdNO_{9.5}$ (%) calcd: C 37.84, H 3.57, N 2.94; found: C 38.85, H 3.408, N 2.98.

MOF 12: $[MV][Cd_2(L_4)_2(L_6)]\cdot 2H_2O$

FT-IR (KBr, cm^{-1}): ν = 3412 (m), 3303 (m), 3266 (m), 3046 (w), 2922 (w), 2855 (w), 1610 (m), 1557 (s), 1373 (s), 1320 (m), 1261 (m), 1026 (w), 962 (w), 837 (w), 786 (w), 732 (w), 576 (w). Elemental analysis for $C_{36}H_{33}Cd_2N_5O_{14}$ (%) calcd: C 43.09, H 3.49, N 6.98; found: C 42.32, H 3.578, N 6.87.

Control experiments:

MOF 13: $[MV][Cd(L_6)Br_{1.25}Cl_{0.75}]$

$Cd(NO_3)_2\cdot 4H_2O$ (54 mg, 0.175 mmol), CL_1 (69 mg, 0.2 mmol), 5-aminoisophthalic acid (18.1 mg, 0.1 mmol), and 2-aminoterephthalic acid (18.1 mg, 0.1 mmol) were added to a mixed solvent of DMF (3.5 mL), methanol (3 mL), ethanol (1 mL), and H_2O (3 mL). The mixture was stirred 5 min until completely dissolved, then sealed in a 20 mL Teflon-lined steel bomb and kept at 85 °C for 72 h. Brown crystals were collected by filtration. FT-IR (KBr, cm^{-1}): ν = 3417 (m), 3305 (m), 3266 (m), 3119 (w), 3045 (w), 1641 (m), 1614 (m), 1557 (vs), 1435 (m), 1371 (s), 1322 (m), 1258 (m), 1023 (w), 966 (w), 842 (w), 802 (w), 778 (m), 733 (w), 576 (w). Elemental analysis for $C_{20}H_{19}CdN_3O_4Cl_{0.75}Br_{1.25}$ (%) calcd: C 39.42, H 3.20, N 6.90; found: C 43.17, H 3.665, N 6.99.

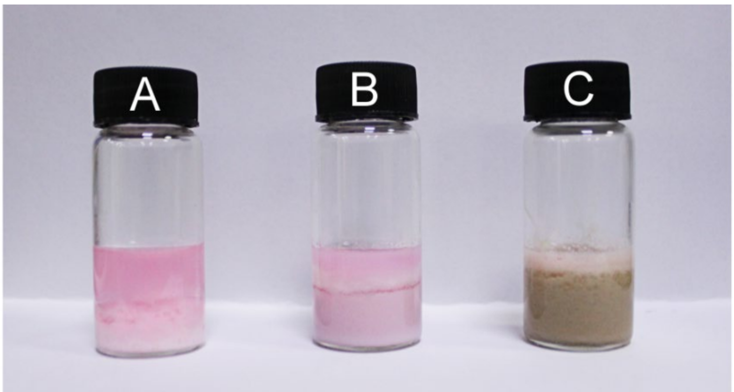

Figure S3. Control experiments of one-pot synthesis of (A) **POMOF 1**, (B) **POMOF 2**, and (C) **POMOF 3**.

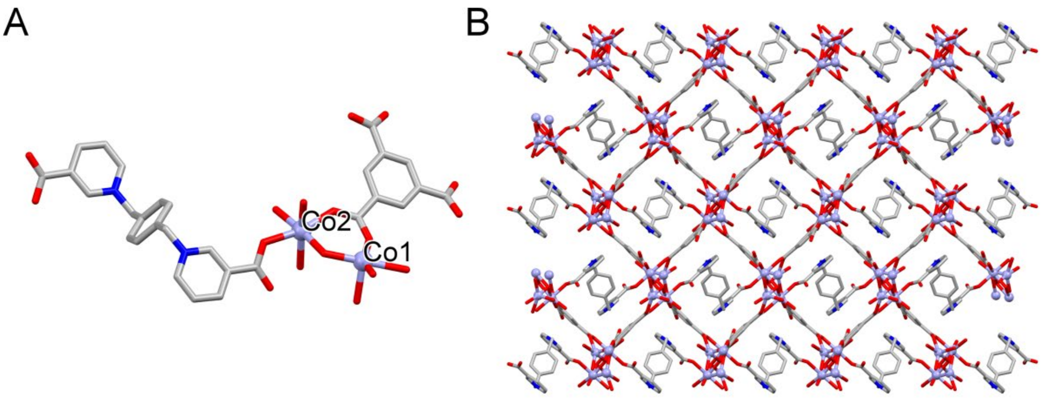

Figure S4. (A) The asymmetric unit and (B) the crystal packing pattern of MOF **1**.

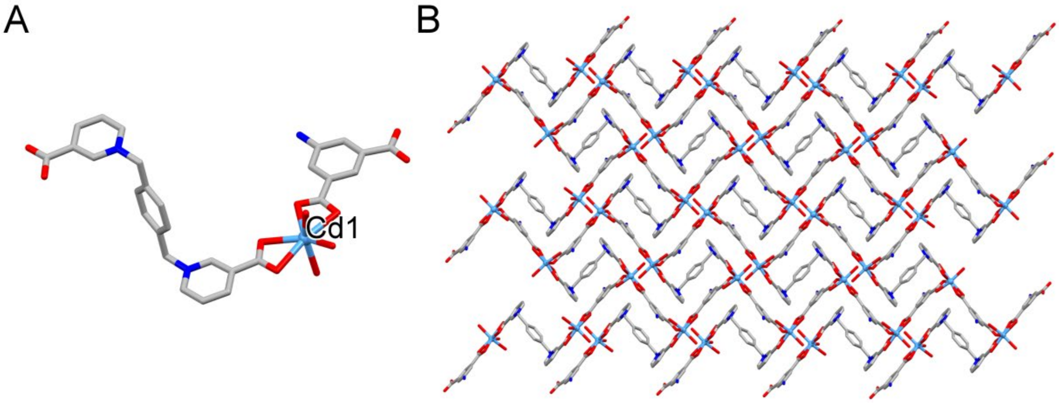

Figure S5. (A) The asymmetric unit and (B) the crystal packing pattern of MOF **2**.

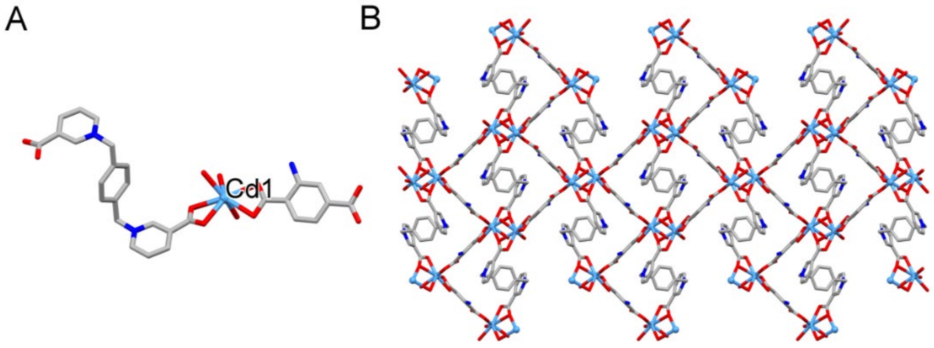

Figure S6. (A) The asymmetric unit and (B) the crystal packing pattern of MOF **3**.

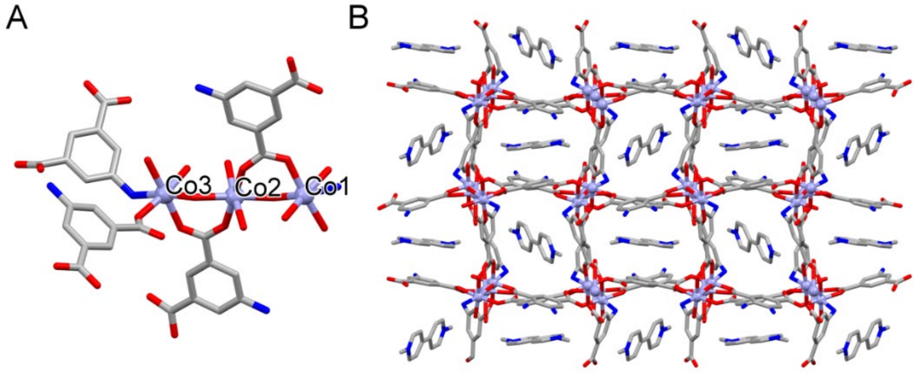

Figure S7. (A) The asymmetric unit and (B) the crystal packing pattern of MOF **4**.

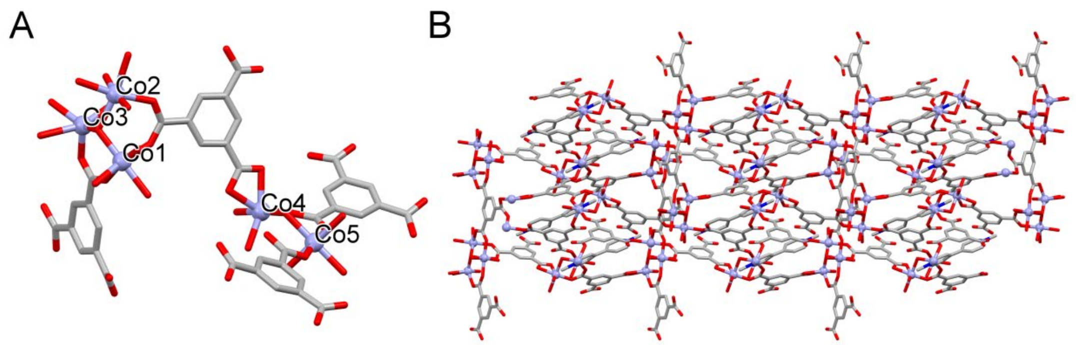

Figure S8. (A) The asymmetric unit and (B) the crystal packing pattern of MOF **5**.

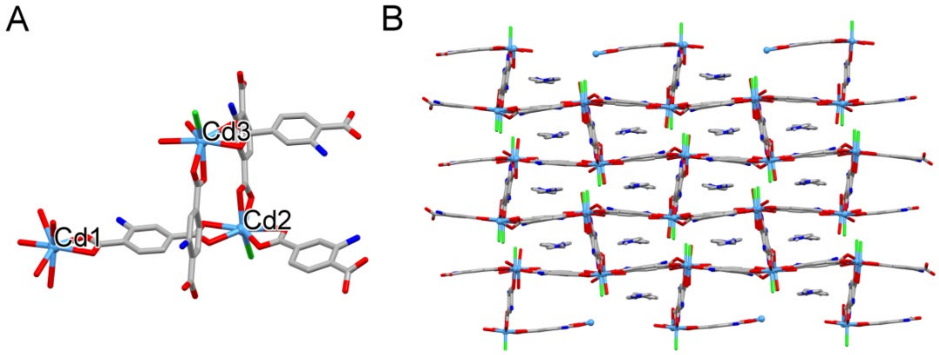

Figure S9. (A) The asymmetric unit and (B) the crystal packing pattern of MOF **6**.

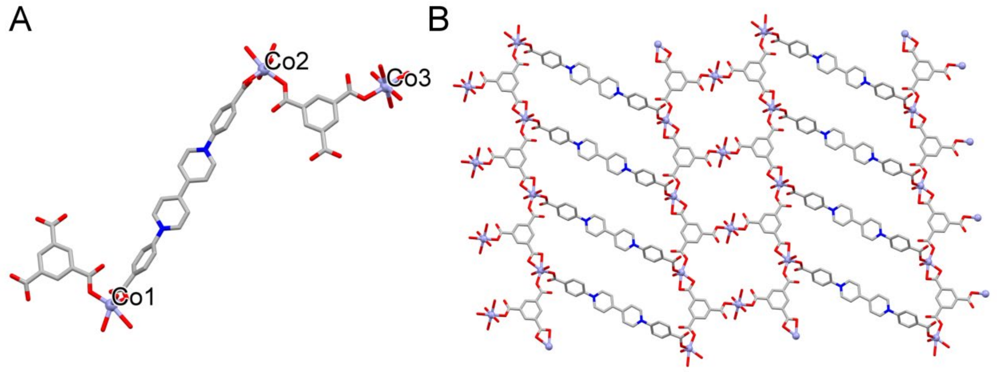

Figure S10. (A) The asymmetric unit and (B) the crystal packing pattern of MOF **7**.

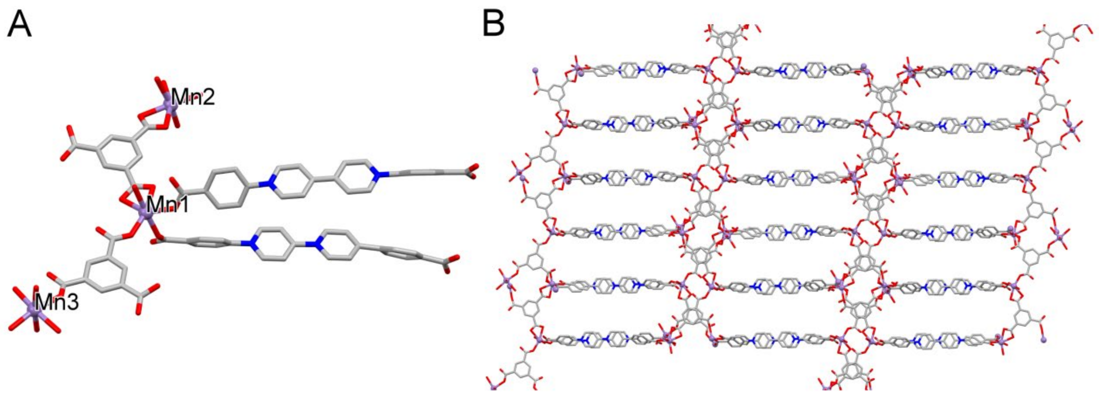

Figure S11. (A) The asymmetric unit and (B) the crystal packing pattern of MOF **8**.

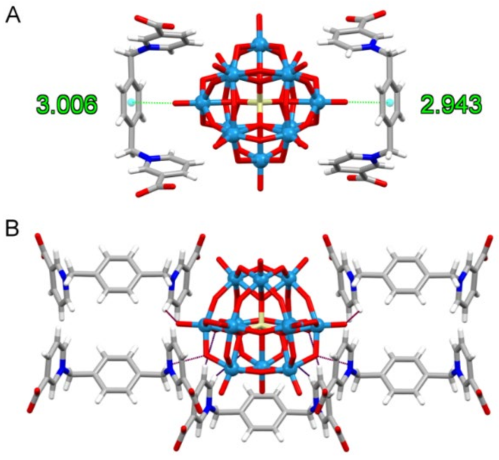

Figure S12. (A) The anion-π interactions and (B) hydrogen bonds between {SiW_{12}} cluster and CL_2 ligands in **POMOF 1**.

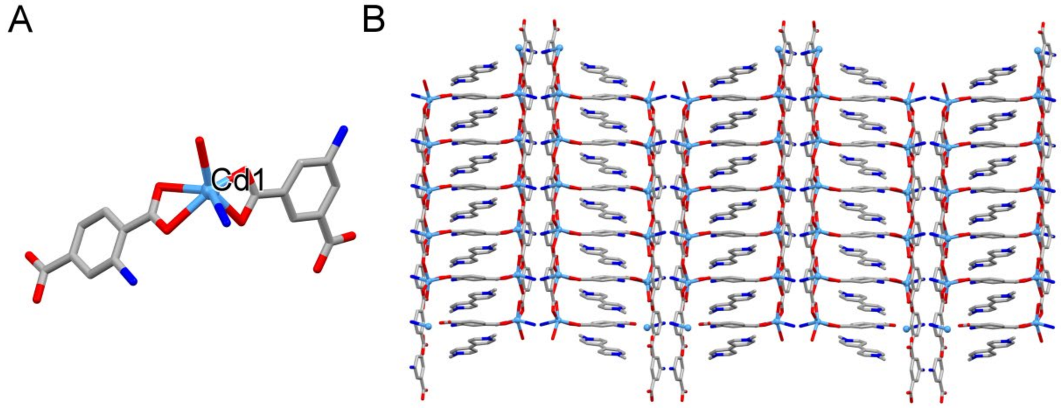

Figure S13. (A) The asymmetric unit and (B) the crystal packing pattern of MOF **12**.

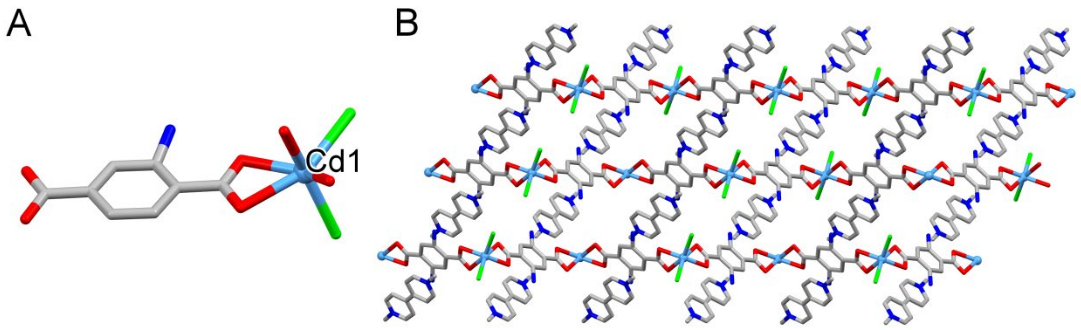

Figure S14. (A) The asymmetric unit and (B) the crystal packing pattern of MOF **13**.

Table S1. Summary of crystal data for MOFs **1**-**4**

	MOF **1**	MOF **2**	MOF **3**	MOF **4**
Formula	$C_{19}H_{22.5}Co_2NO_{14.25}$	$C_{18}H_{17}CdN_2O_8$	$C_{18}H_{15}CdN_2O_7$	$C_{41}H_{41}Co_3N_{5.5}O_{21}$
F.W.	610.74	501.73	483.72	1123.58
Cryst.Syst.	monoclinic	monoclinic	monoclinic	monoclinic
Space group	$P2_1/n$	$P2_1/n$	$P2_1/c$	$P2_1/n$
a/Å	12.1003(3)	8.4173(3)	8.5287(4)	14.1160(4)
b/Å	16.3368(3)	13.2921(3)	17.8713(9)	19.5199(7)
c/Å	12.3245(2)	16.6093(8)	11.0843(5)	17.3852(4)
α (°)	90	90	90	90
β (°)	94.357(2)	103.777(4)	97.415(4)	93.588(2)
γ (°)	90	90	90	90
V/Å^3	2429.27(8)	1804.84(12)	1675.33(14)	4781.0(2)
Z	4	4	4	4
ρ	1.670	1.846	1.918	1.561
R_1	0.0450	0.0249	0.0739	0.0878
wR_2	0.1325	0.0611	0.2994	0.2733

Table S2. Summary of crystal data for MOFs **5**-**8**

	MOF **5**	MOF **6**	MOF **7**	MOF **8**
Formula	$C_{55}H_{73}Co_5N_4O_{41.5}$	$C_{60}H_{69.5}Cd_3N_{8.5}O_{27.5}Cl$	$C_{42}H_{44}Co_3N_2O_{27}$	$C_{66}H_{67}Mn_3N_4O_{34.5}$
F.W.	1748.82	1722.39	1185.60	1633.05
Cryst.Syst.	monoclinic	monoclinic	triclinic	monoclinic
Space group	$P2_1$	$P2_1$/n	P-1	$P2_1$/n
a/Å	11.1688(2)	13.0031(3)	9.955(2)	18.382(5)
b/Å	16.0722(2)	20.1904(4)	10.250(2)	13.676(4)
c/Å	20.2135(3)	26.5700(6)	10.974(2)	28.013(8)
α (°)	90	90	92.10(3)	90
β (°)	92.589(1)	90.035(2)	97.02(3)	101.495(5)
γ (°)	90	90	100.19(3)	90
V/Å^3	3624.77(10)	6975.6(3)	1091.9(4)	6901(3)
Z	2	4	1	4
ρ	1.602	1.640	1.803	1.572
R_1	0.0557	0.0617	0.0334	0.1102
wR_2	0.1566	0.1837	0.0770	0.3424

Table S3. Summary of crystal data for **POMOFs 1-3**

	**POMOF 1**	**POMOF 2**	**POMOF 3**
Formula	$C_{49}H_{69}Co_2SiW_{12}N_7O_{59}$	$C_{26}H_{45}CoCrMo_6N_4O_{34}$	$C_{28}H_{56}Cr_{1.67}Mo_6N_4O_{38}Na$
F.W.	4052.26	1644.23	1742.08
Cryst.Syst.	monoclinic	triclinic	triclinic
Space group	*Cm*	*P*-1	*P*-1
a/Å	14.2325(4)	10.1292(3)	9.9536(3)
b/Å	18.2760(6)	11.3306(3)	11.7858(5)
c/Å	16.4709(6)	12.3308(4)	12.3214(4)
α (°)	90	114.244(3)	114.368(4)
β (°)	99.574(3)	94.359(3)	91.155(3)
γ (°)	90	92.555(2)	93.198(3)
V/Å^3	4224.6(2)	1282.18(7)	1313.14(9)
Z	2	1	1
ρ	3.186	2.129	2.203
R_1	0.0449	0.0404	0.0684
wR_2	0.1339	0.1105	0.1904

Table S4. Summary of crystal data for MOFs **9**-**11**

	MOF **9**	MOF **10**	MOF **11**
Formula	$C_{78}H_{98}Cd_4Co_3N_4O_{62}$	$C_{48}H_{54}Cd_4CoN_2O_{38}$	$C_{15}H_{17}CdNO_{9.5}$
F.W.	1355.03	1775.46	475.71
Cryst.Syst.	monoclinic	monoclinic	monoclinic
Space group	$P2_1/c$	$P2_1/c$	$C2/c$
a/Å	9.7515(2)	10.1709(2)	13.1952(9)
b/Å	31.2668(8)	10.6652(2)	15.5766(8)
c/Å	16.3206(3)	29.4488(5)	16.5993(9)
α (°)	90	90	90
β (°)	103.502(2)	98.362(2)	108.983(6)
γ (°)	90	90	90
V/Å^3	4838.59(19)	3160.49(10)	3226.2(3)
Z	2	2	8
ρ	1.860	1.866	1.959
R_1	0.0573	0.0476	0.0824
wR_2	0.1288	0.1388	0.1765

Table S5. Summary of crystal data for MOFs **12** and **13**

	MOF **12**	MOF **13**
Formula	$C_{36}H_{33}Cd_2N_5O_{14}$	$C_{20}H_{19}CdN_3O_4Cl_{0.75}Br_{1.25}$
F.W.	984.47	604.26
Cryst.Syst.	triclinic	orthorhombic
Space group	*P*-1	*P*ccn
a/Å	8.1024(11)	8.0601(2)
b/Å	8.1286(11)	14.6116(4)
c/Å	15.3665(17)	18.5311(8)
α (°)	101.212(11)	90
β (°)	102.078(11)	90
γ (°)	102.173(12)	90
V/Å^3	936.8(2)	2182.43(12)
Z	1	4
ρ	1.745	1.839
R_1	0.0945	0.0599
wR_2	0.2772	0.1703

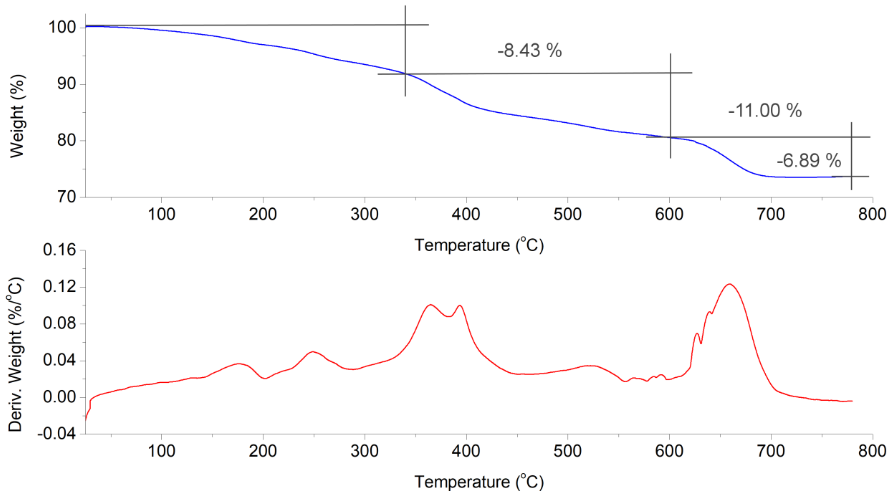

Figure S16. The TGA and DTG curves of **POMOF 1**.

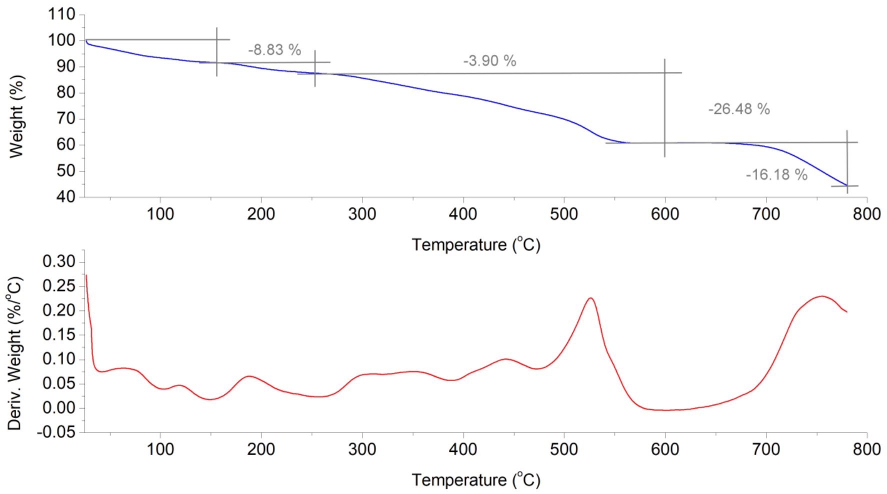

Figure S17. The TGA and DTG curves of **POMOF 2**.

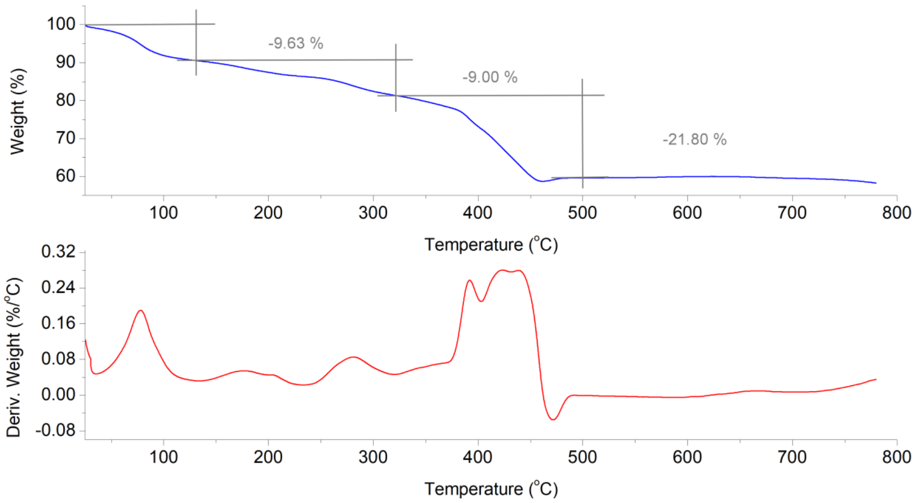

Figure S18. The TGA and DTG curves of **POMOF 3**.

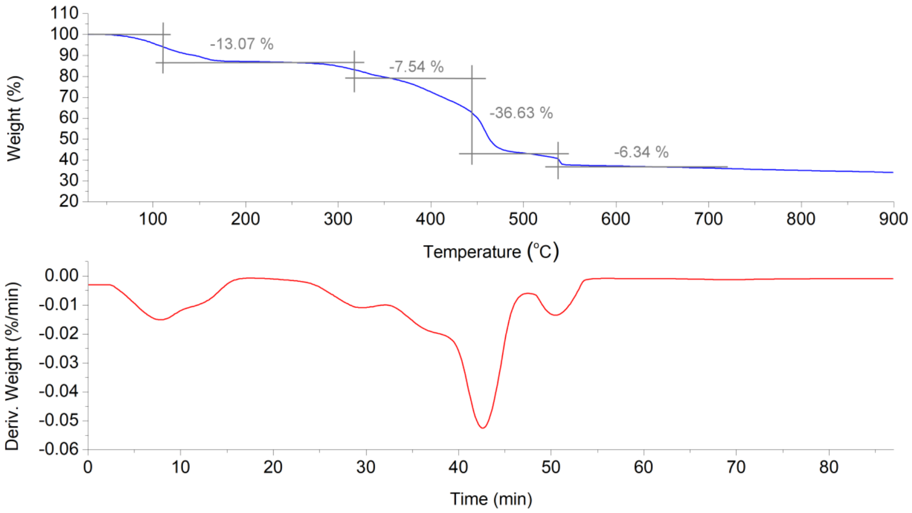

Figure S19. The TGA and DTG curves of MOF **1**.

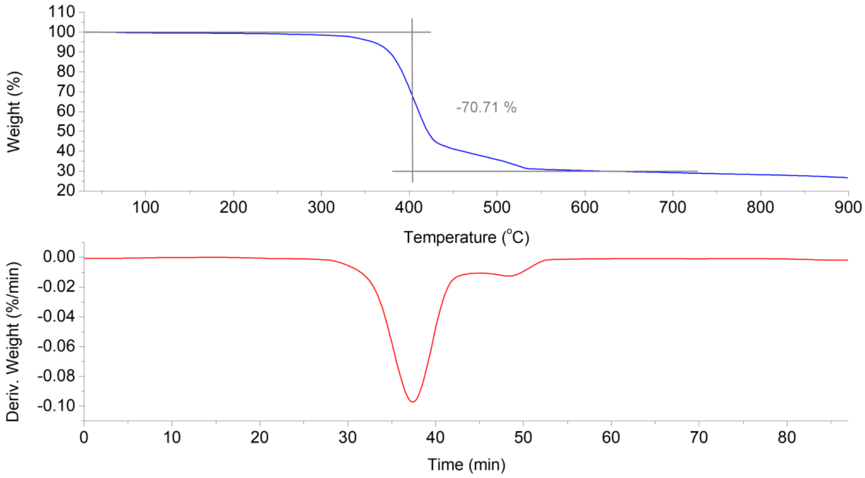

Figure S20. The TGA and DTG curves of MOF **2**.

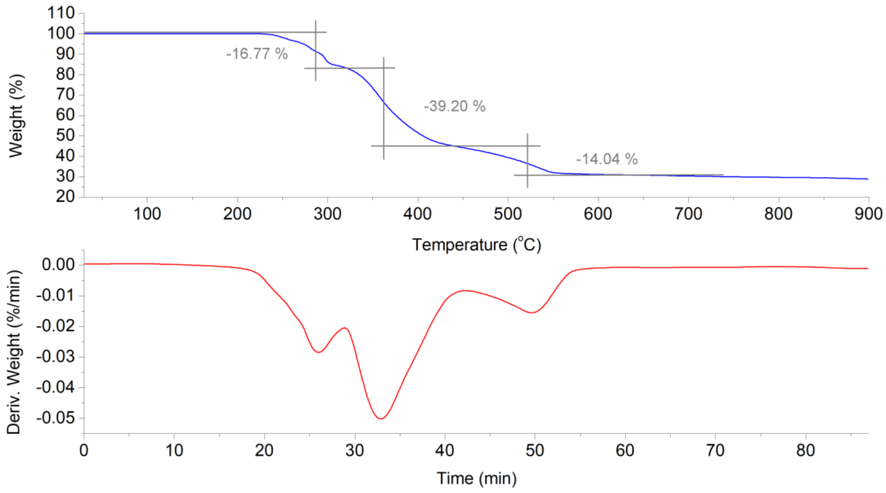

Figure S21. The TGA and DTG curves of MOF **3**.

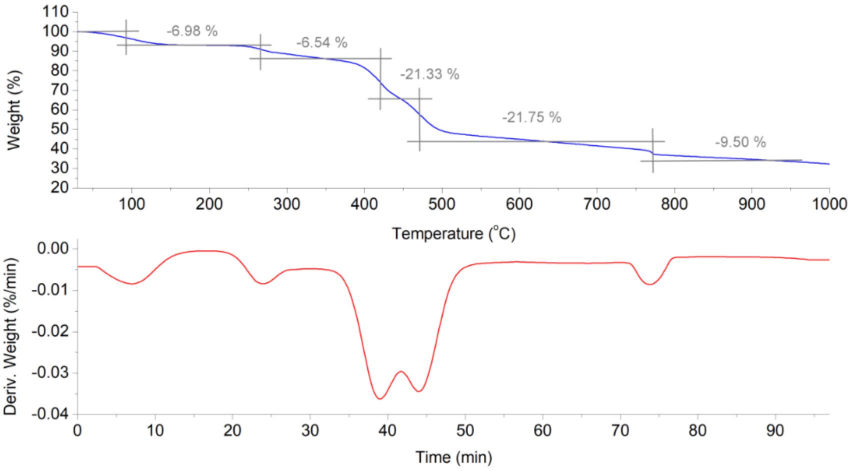

Figure S22. The TGA and DTG curves of MOF **4**.

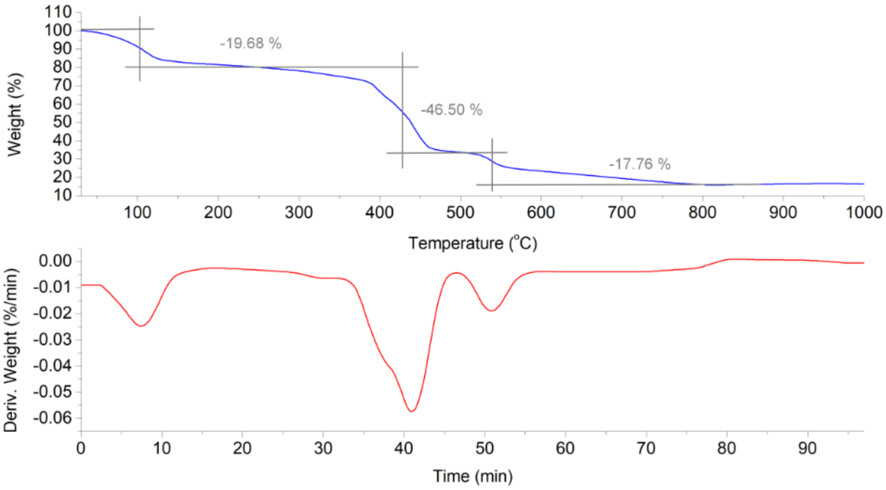

Figure S23. The TGA and DTG curves of MOF **5**.

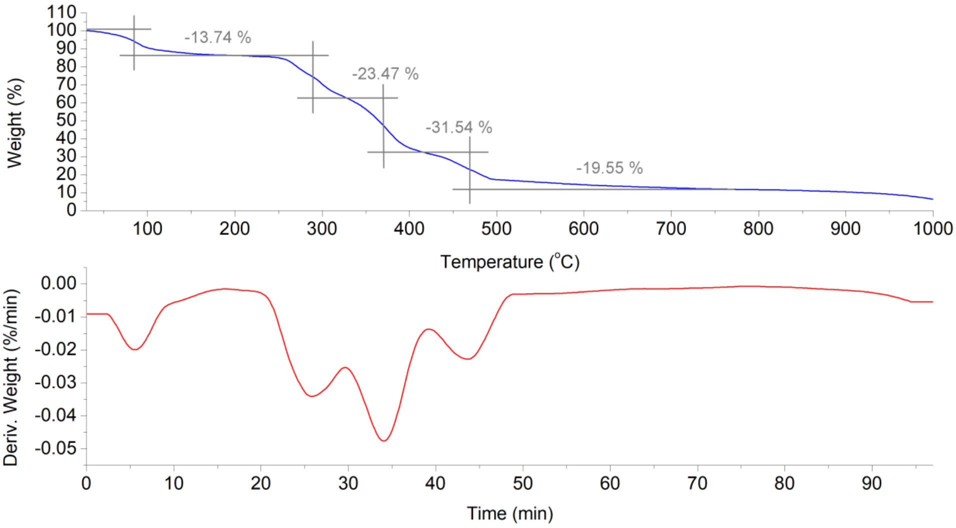

Figure S24. The TGA and DTG curves of MOF **6**.

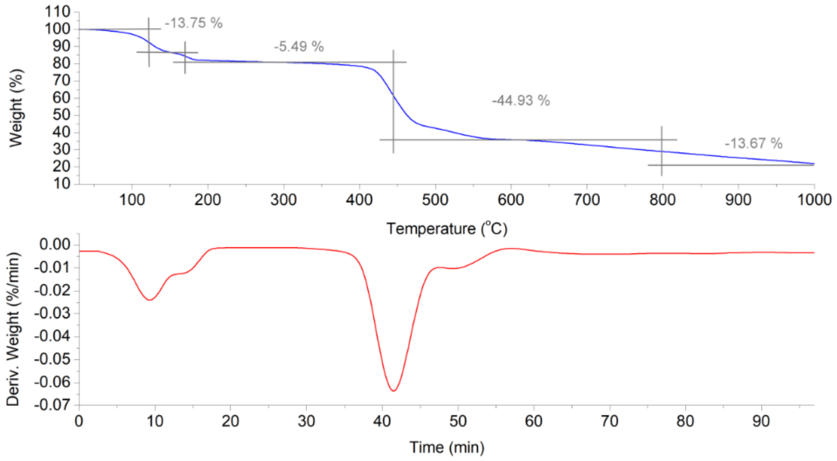

Figure S25. The TGA and DTG curves of MOF **7**.

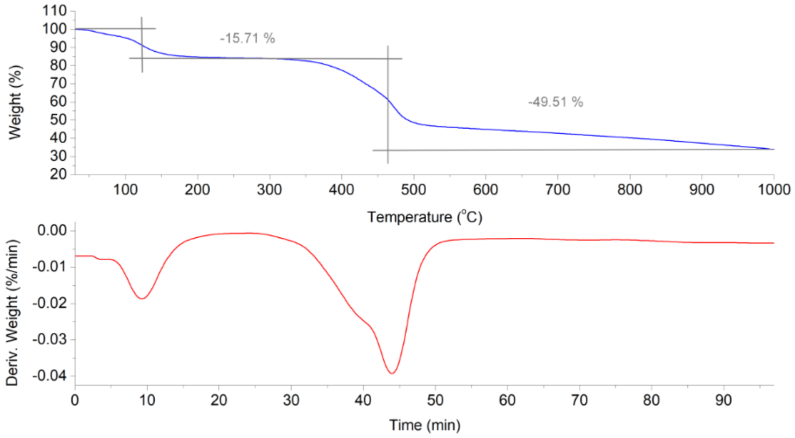

Figure S26. The TGA and DTG curves of MOF **8**.

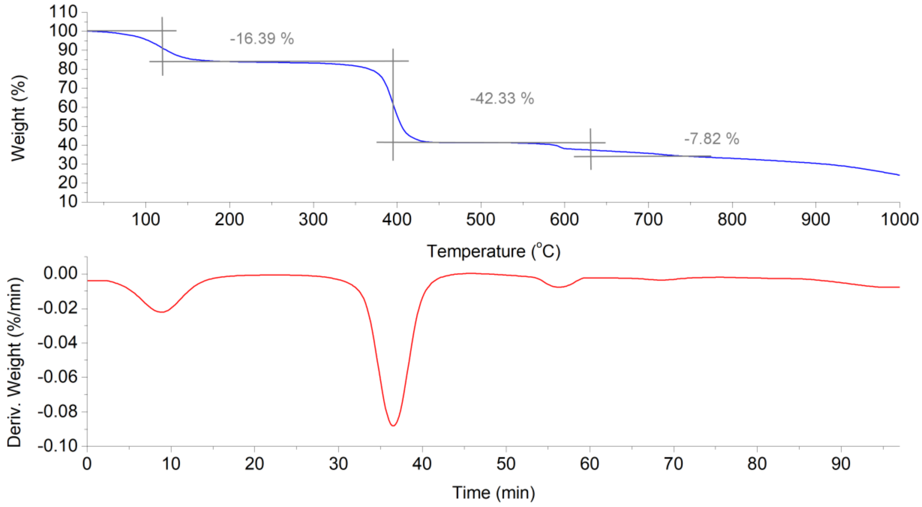

Figure S27. The TGA and DTG curves of MOF **9**.

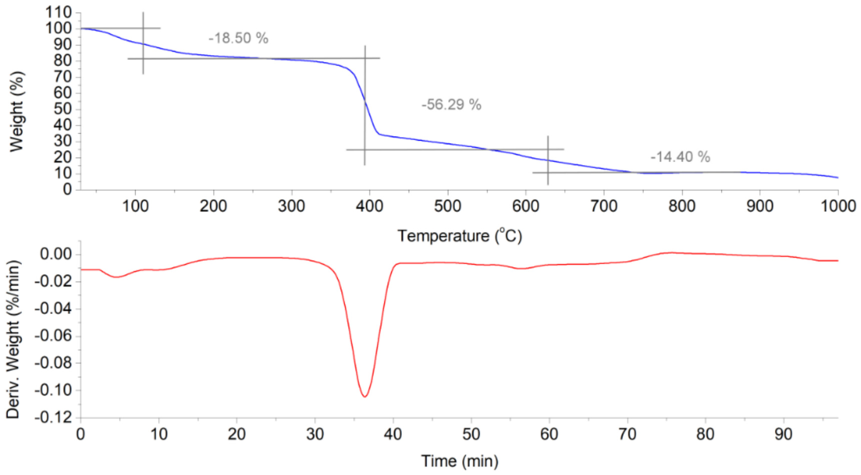

Figure S28. The TGA and DTG curves of MOF **10**.

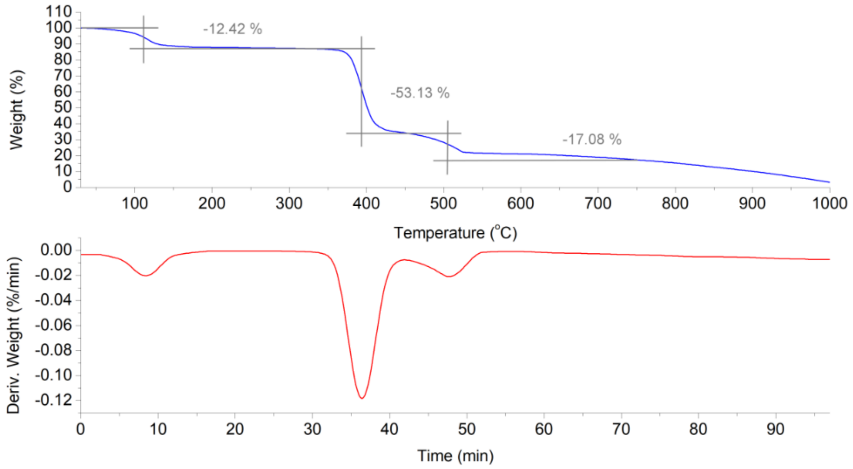

Figure S29. The TGA and DTG curves of MOF **11**.

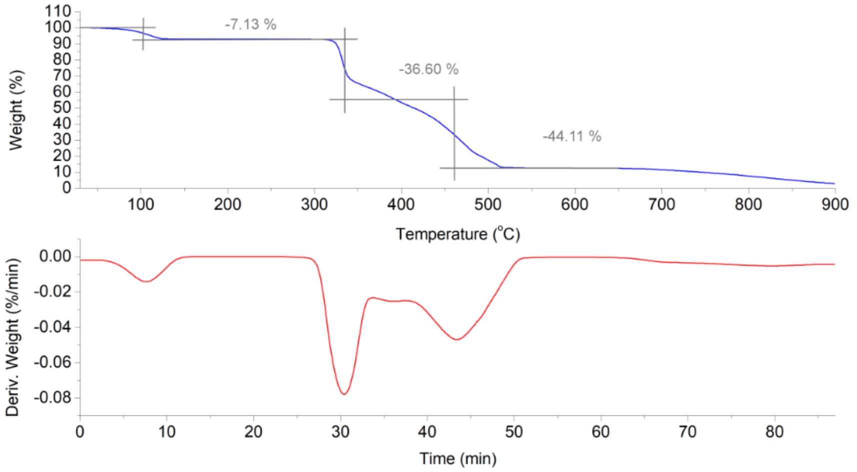

Figure S30. The TGA and DTG curves of MOF **12**.

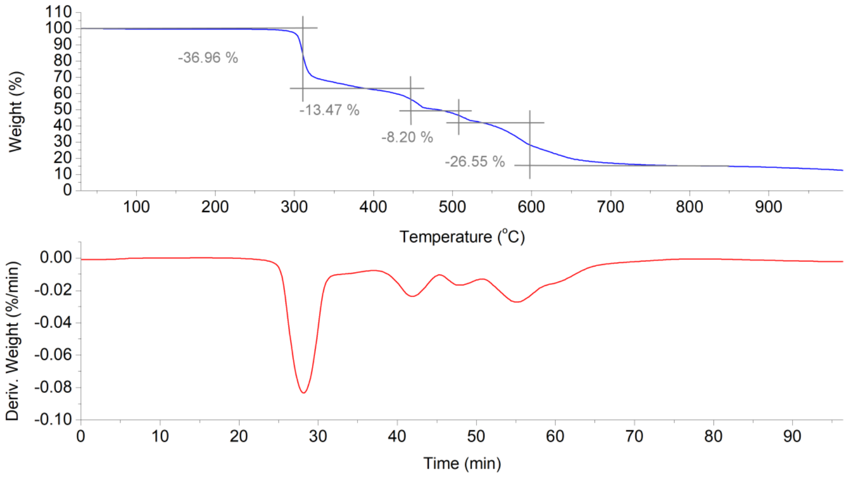

Figure S31. The TGA and DTG curves of MOF **13**.

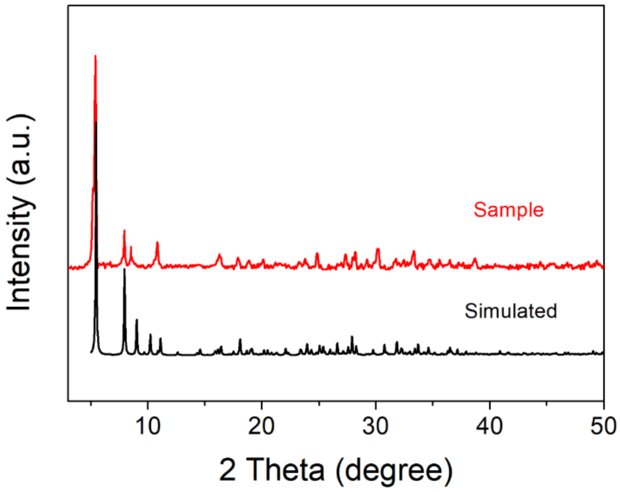

Figure S32. The powder X-ray diffraction patterns of **POMOF 1**.

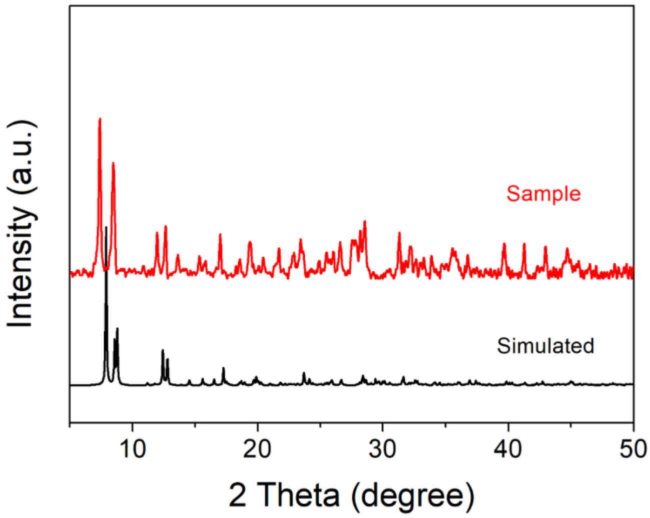

Figure S33. The powder X-ray diffraction patterns of **POMOF 2**.

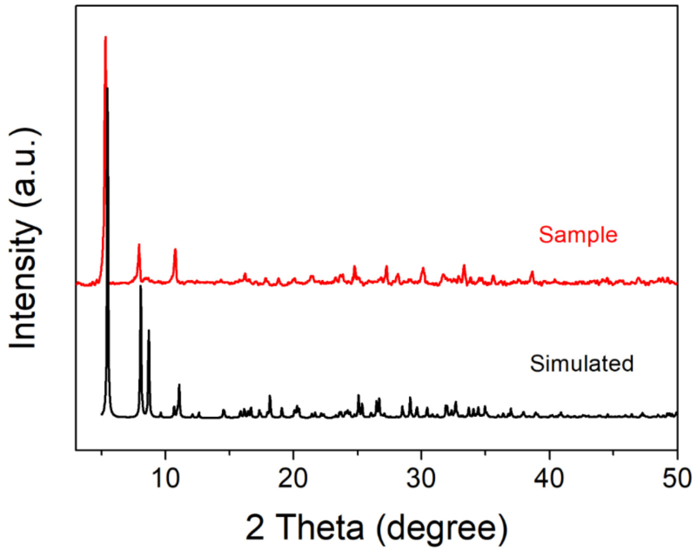

Figure S34. The powder X-ray diffraction patterns of **POMOF 3**.

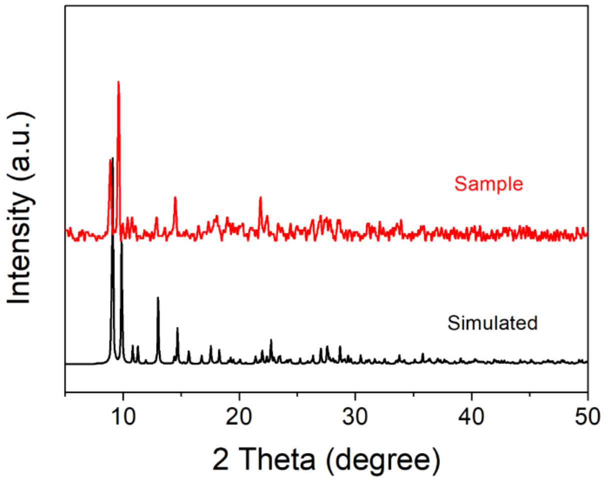

Figure S35. The powder X-ray diffraction patterns of MOF **1**.

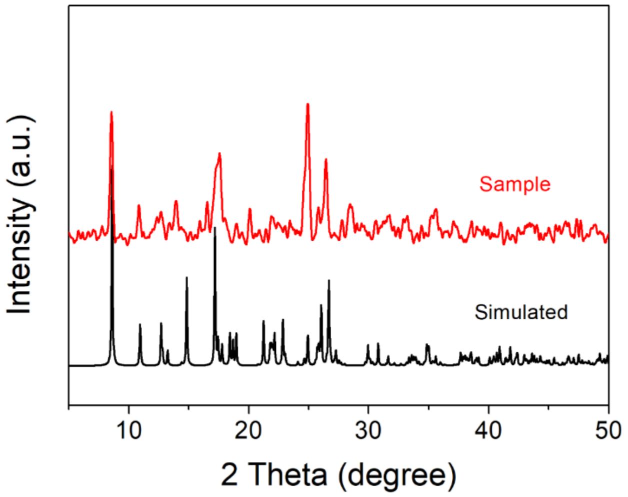

Figure S36. The powder X-ray diffraction patterns of MOF **2**.

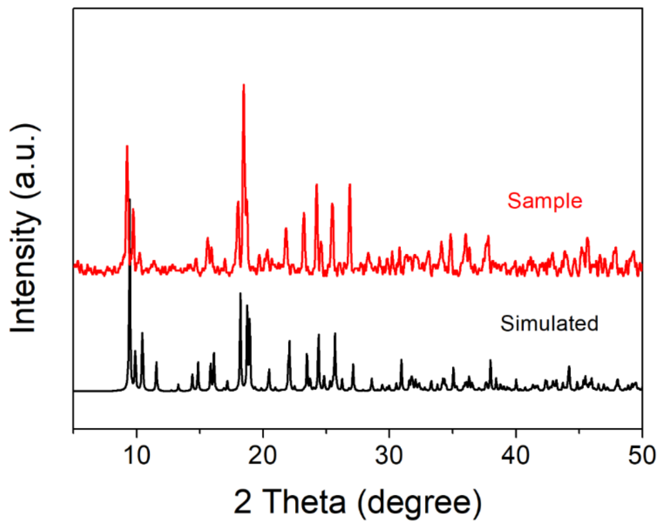

Figure S37. The powder X-ray diffraction patterns of MOF **3**.

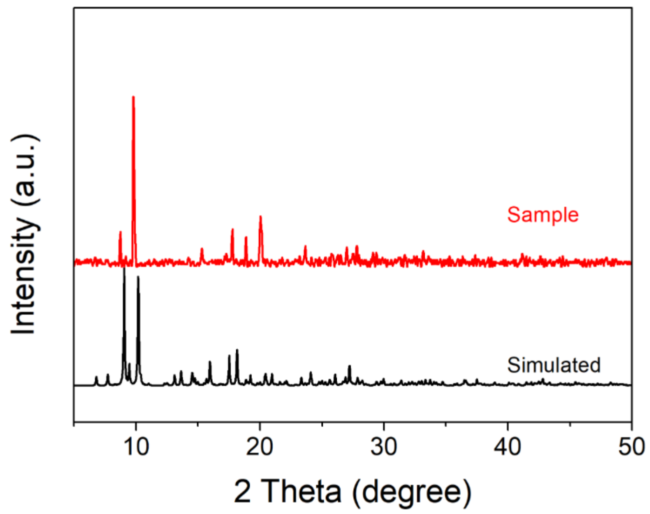

Figure S38. The powder X-ray diffraction patterns of MOF **4**.

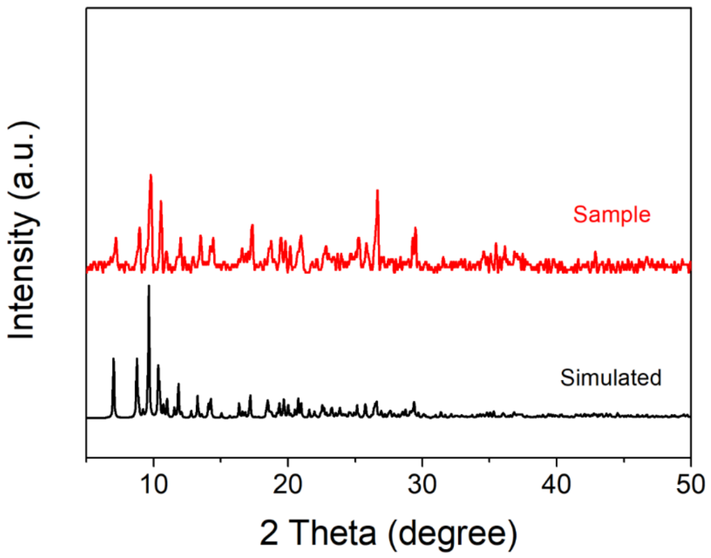

Figure S39. The powder X-ray diffraction patterns of MOF **5**.

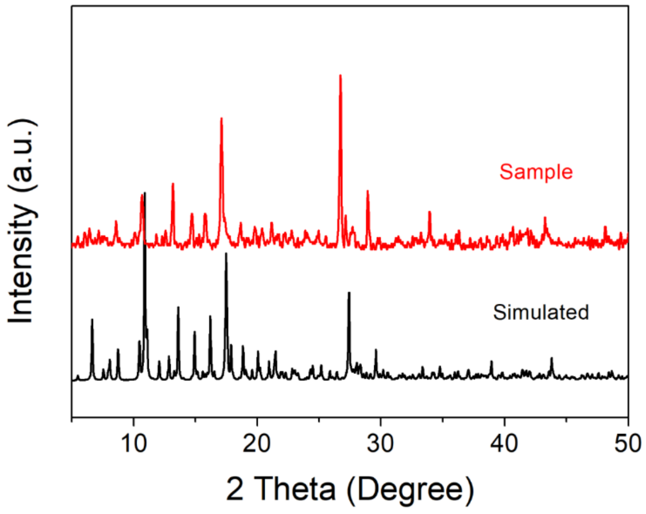

Figure S40. The powder X-ray diffraction patterns of MOF **6**.

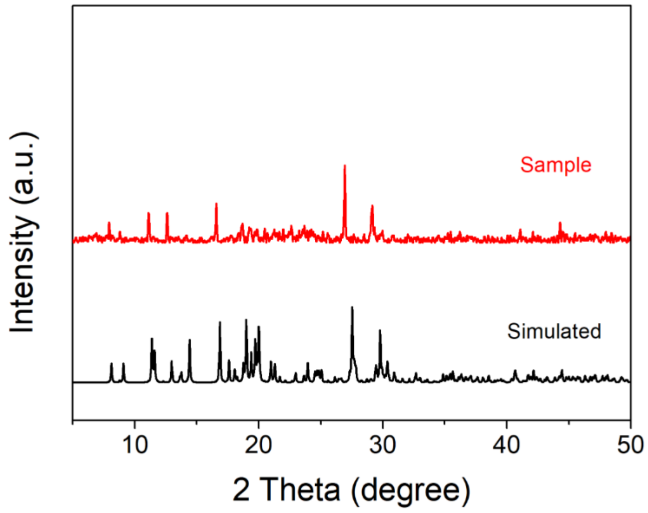

Figure S41. The powder X-ray diffraction patterns of MOF **7**.

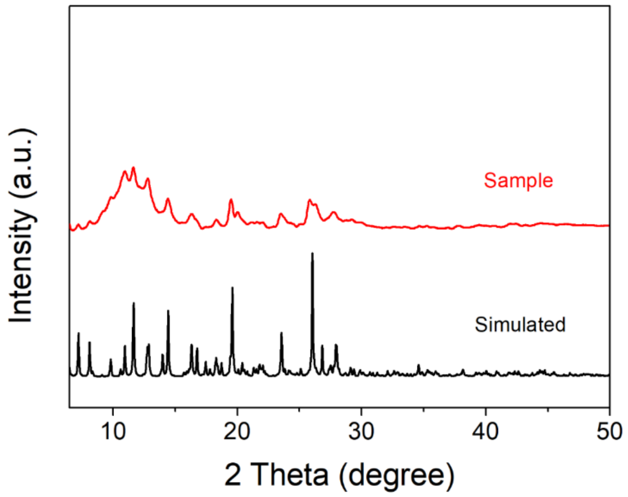

Figure S42. The powder X-ray diffraction patterns of MOF **8**. MOF **8** was unstable in the air and the PXRD data was obtained on an Oxford Gemini diffractometer.

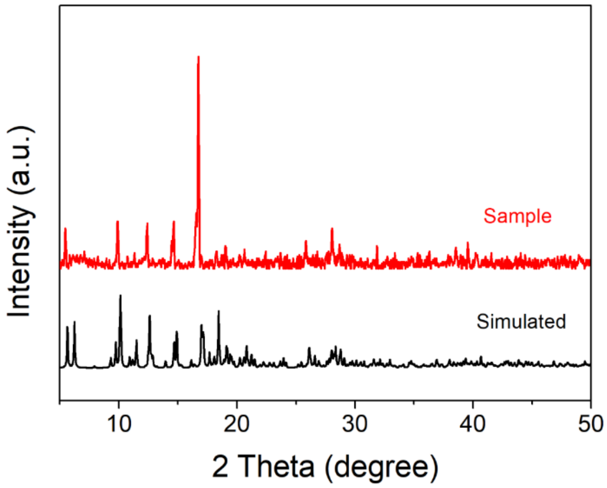

Figure S43. The powder X-ray diffraction patterns of MOF **9**.

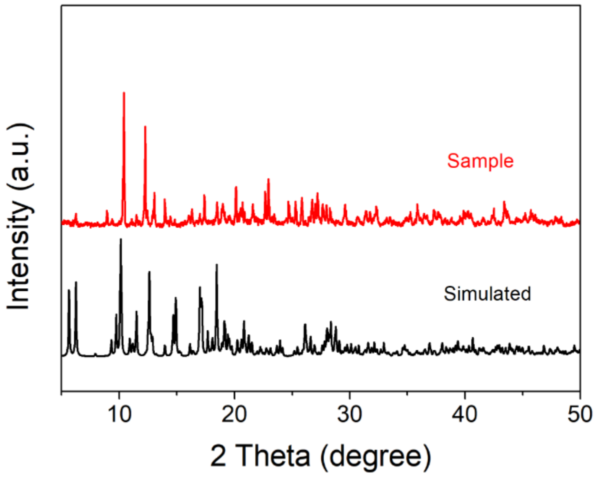

Figure S44. The powder X-ray diffraction patterns of MOF **10**.

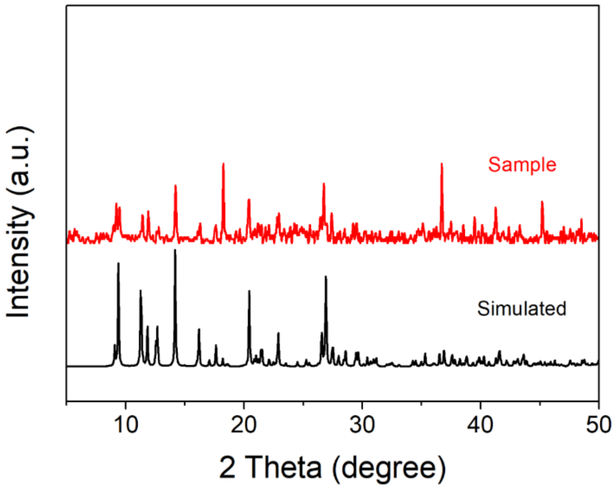

Figure S45. The powder X-ray diffraction patterns of MOF **11**.

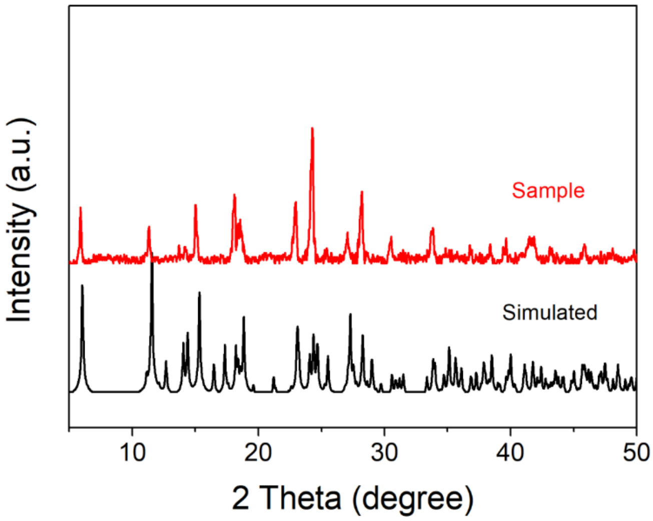

Figure S46. The powder X-ray diffraction patterns of MOF **12**.

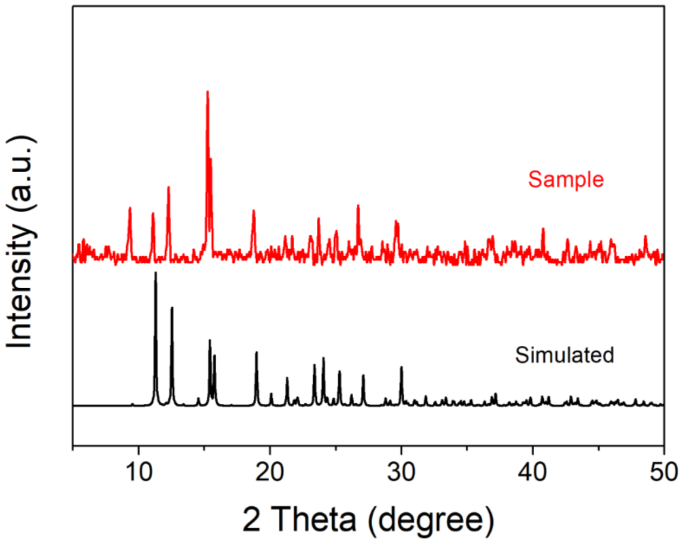

Figure S47. The powder X-ray diffraction patterns of MOF **13**.

References

[1]. G. M. Sheldrick, *Acta Cryst. A*, **2015**, *71*, 3.

[2]. G. M. Sheldrick, *Acta Cryst. C*, **2015**, *71*, 3.

[3]. A. D. Phillips, Z. Fei, W. H. Ang, R. Scopelliti and P. J. Dyson, *Cryst. Growth. Des.,* **2009**, *9*, 1966.

[4]. F. K. Zheng, A. Q. Wu, Y. Li, G. C. Guo, J. S. Huang, *Chin. J. Struct. Chem.*, **2005**, *8*, 940.

[5]. J. J. Liu, Y. F. Guan, M. J. Lin, C. C. Huang and W. X. Dai, *Cryst. Growth Des.,* **2015**, *15*, 5040.
